# Wound Botulism in the Setting of Pregnancy: A Literature Review and Case Report

**DOI:** 10.7759/cureus.29273

**Published:** 2022-09-17

**Authors:** Henrik Ghantarchyan, Alexander T Phan, Janie Hu, Kunal Thaker, Inessa Dombrovsky, Kristina Roloff, Sarkis Arabian

**Affiliations:** 1 Internal Medicine, Arrowhead Regional Medidcal Center, Colton, USA; 2 Internal Medicine, California University of Science and Medicine, Colton, USA; 3 Internal Medicine, Arrowhead Regional Medical Center, Colton, USA; 4 Women's Health, Arrowhead Regional Medical Center, Colton, USA; 5 Critical Care, Arrowhead Regional Medical Center, Colton, USA

**Keywords:** neuromuscular weakness, respiratory failure, botulism, equine antitoxin, black tar heroin, pregnancy, botulinum toxin

## Abstract

Botulism is a rare neuroparalytic illness caused by *Clostridium botulinum* that can manifest as a descending flaccid paralysis, progressing from cranial neuropathies to respiratory failure. Wound botulism, constituting a minority of cases, is majorly associated with the injection of black tar heroin (BTH) in the western United States. A patient population of particular concern is pregnant women, who may experience a more severe course due to the physiological changes that occur in pregnancy. Because botulism in pregnancy lacks pathognomic features, physicians should maintain a high clinical suspicion when faced with a pregnant patient with neurological symptoms and a history of BTH use. Here, we report the case of a 25-year-old G3P1A1 female with a history of BTH use who presented with cranial neuropathies and respiratory insufficiency.

## Introduction

​​Botulism is a rare neuroparalytic illness caused by* Clostridium botulinum*, a gram-positive, spore-forming, obligate anaerobic rod. The heat-labile botulinum neurotoxin (BoNT) binds presynaptically to the neuromuscular junction and prevents acetylcholine release, resulting in descending flaccid paralysis [1,2]. Initial presentation often involves cranial neuropathies, which can include ptosis, facial muscle weakness, diplopia, blurred vision, dysphagia, and dysarthria. Subsequent respiratory involvement may occur due to muscle weakness and diaphragm paralysis leading to hypoxia, dyspnea, and respiratory failure [1]. BoNT can also cause autonomic disturbances, such as dry mouth, constipation, and urinary retention. However, notably, sensation and reflexes remain intact [2].

There are seven types of BoNTs, types A through G, of which human illnesses typically arise via serovars A, B, and E [3]. There are many routes of BoNT exposure, but the majority of transmission comes from intestinal colonization, inhalation, or wound contamination. Wound botulism occurs from wound contamination with *C. botulinum* spores, which germinate and produce BoNT under anaerobic conditions. Commonly seen in crush injuries, gunshot wounds, and open fractures, the first reported case of injection drug use was in 1982 in New York City [4]. Since 2016, there has been a marked increase in the number of wound botulism cases, with 82% of the country's reported cases isolated in California, where the use of BTH is common [3,5].

BTH is a black-colored form of heroin that is gummy in consistency, mainly produced in Mexico, and diluted with shoe polish and dirt [5]. Although wound botulism accounts for less than 10% of cases annually [3], physicians should maintain a high degree of clinical suspicion when evaluating patients with neuroparalytic symptoms and a history of injection drug use [6]. This is especially important in the western United States, where BTH is widely used [7]. A patient population of particular concern is pregnant women, who may experience a more severe course and poor outcomes [8]. In caring for pregnant patients presenting with neurological symptoms and a history of BTH use, physicians should consider botulism as a differential for early diagnosis and treatment [9]. Here, we report the case of a 25-year-old G3P1A1 female with a history of BTH use who presented with cranial neuropathies and respiratory insufficiency.

## Case presentation

The patient is a 25-year-old G3P1A1 female with a past medical history of polysubstance abuse who presented to the emergency department by ambulance for hypoxia and a suspected heroin overdose. The patient was noted to have an oxygen saturation of 70% in the field, which improved to 95% with supplemental oxygen via a non-rebreather mask. She was also given naloxone 4 mg intramuscularly en route to the hospital. On arrival, the patient reported subjective dyspnea, generalized myalgias, weakness, and dysphagia. Of note, she also admitted to daily intramuscular heroin, which she described as “black tar heroin” (BTH). She denied any other past medical history, including neurological issues. The patient’s initial vital signs included a body temperature of 98.6 F, pulse rate of 130 bpm, respiratory rate of 25 breaths per minute, blood pressure of 147/98 mmHg, and oxygen saturation of 100% on the non-rebreather mask. Physical exam was notable for right-sided ptosis, right-sided facial droop, bulbar weakness, and scarring on the patient’s bilateral buttocks and arms.

**Figure 1:**
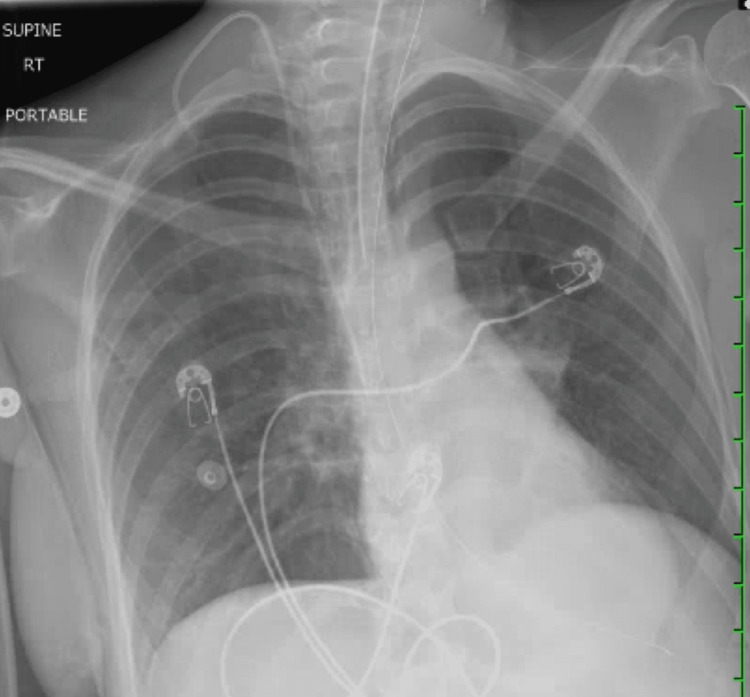
Chest radiograph demonstrating endotracheal tube.

The patient’s initial laboratory findings are shown in Table 1, demonstrating leukocytosis without bandemia and anemia. Her urine drug screen was positive for opiates. Computed tomography (CT) of her brain did not reveal any acute intracranial abnormalities. The chest x-ray was also unremarkable (Figure 1). An ultrasound of the pelvis revealed a single intrauterine pregnancy with a gestational age of 14 weeks and 0 days. Given the patient’s clinical presentation and history of intramuscular heroin injection, there was a high degree of suspicion of clinical botulism. The United States Centers for Disease Control and the California Department of Public Health were notified for immediate acquisition of the botulinum antitoxin. Upon delivery, the patient was subsequently administered the equine serum heptavalent botulism antitoxin intravenously (IV).

**Table 1:** Significant laboratory results from initial presentation to the hospital. *μL = microliter, g = gram, dL = deciliter, mEq = milliequivalent, mmol = millimole, mg = milligram

	WBC (cells/μL)	Hemoglobin (g/dL)	RBC (million cells/μL)	Hematocrit (%)	Platelets (cells/μL)	Segmented Neutrophils (%)	Band Neutrophils (%)
Reference Values	N=4,300-11,000	N=13-17	N=4.5-5.9	N=41-53	N=120,000-360,000	N=55-70	N=2-5
Measured Values	18,200	10.5	4.39	33.5	360,000	83	2
	Sodium (mEq/dL)	Potassium (mEq/dL)	Chloride (mEq/dL)	Carbon Dioxide (mmol/L)	BUN (mg/dL)	Creatinine (mg/dL)	Calcium (mg/dL)
Reference Values	135-148	3.5-5.5	98-110	24-34	8-20	0.5-1.5	8.4-10.2
Measured Values	133	4.2	102	20	12	0.2	9.3

After administration of the antitoxin, no adverse effects were observed, however, the patient continued to experience bulbar weakness, so the decision was made to transfer the patient to the ICU for close monitoring given the concern for impending airway compromise. While being observed, the patient’s respiratory mechanics were serially measured, and given her weak mechanics and pooling of oral secretions, the decision was made to intubate the patient. Consequently, the patient was sedated, orotracheally intubated, and placed on a mechanical ventilator. Blood cultures did not reveal any microbial organisms. Due to concerns for aspiration pneumonia and to cover for any wound botulism, ampicillin/sulbactam 3 g IV was administered every 6 hours. The maternal-fetal medicine specialists were consulted to assist in obstetrics management for the patient. Fetal heart rate remained within normal limits during the patient’s hospitalization. Over the patient’s hospital course, her leukocytosis resolved, and she did not experience any further complications. While in the ICU, the patient was on minimal sedation and serial mechanics were checked with slow improvement observed. After about 48 hours, the patient was deemed appropriate for extubation and successfully extubated. She subsequently left the hospital against medical advice and has not returned to the hospital since. As a result, we were unable to follow her after hospital discharge.

## Discussion

Botulism is characterized by a descending motor neuropathy that spares the sensory pathways and deep tendon reflexes, manifesting as blurred vision, ptosis, dysphagia, dysarthria, facial muscle weakness, limb paralysis, and respiratory compromise [1,2,4,5,8,9]. It is classified into six categories: wound, food, enteric, inhaled, iatrogenic, and infant-borne, with the wound as the fifth most common type [4,9]. When considering botulism, it is important to rule out other neuromuscular diseases including Guillain-Barré syndrome (GBS), myasthenia gravis (MG), Eaton-Lambert syndrome, stroke, and opioid overdose [5,8]. Botulism warrants timely treatment with IV administration of the equine heptavalent botulism antitoxin. Early administration of this antidote within 72 hours of initial presentation has been shown to decrease morbidity and mortality [8,9]. Due to its low prevalence, botulism may be under-recognized unless actively considered. A presumptive diagnosis should be made clinically for early delivery of antitoxin, and later confirmed by serum botulinum toxin testing [2,3,8].

​​Physiologic changes during pregnancy result in decreased pulmonary functional residual capacity, decreased total lung capacity, decreased residual volume, decreased expiratory reserve volume, and increased oxygen consumption; therefore, respiratory failure often occurs more rapidly in pregnant patients [8]. Early diagnosis and treatment of botulism may help prevent adverse fetal outcomes associated with hypoxia in pregnancy, especially in patients with pertinent risk factors for botulism. Since clinical signs for pregnant and non-pregnant patients are typically indistinguishable, a high degree of suspicion is warranted. This is especially important as the adverse effects of botulism may be devastating to the mother and fetus [9]. Thus, in addition to consulting an airway specialist to monitor for airway compromise and respiratory failure, it is also important to consider consulting a maternal-fetal medicine specialist to decrease the risk of fetal demise and further complications [5,9].

It is common for providers to mistake the early stages of botulism with pregnancy-related findings or other neurologic disorders [8]. After we determined that the patient’s presenting symptoms were not consistent with normal pregnancy findings, our differential diagnoses broadened. These differentials included MG, GBS, and Eaton-Lambert syndrome. We essentially ruled out opioid overdose, as the patient had been given naloxone and experienced worsening neurologic symptoms over 24 hours after hospital admission. MG was of concern due to its similarities with botulism; however, it was excluded, as the patient’s paralysis was observed to be in a descending manner, manifesting as ptosis, bulbar weakness, and facial droop with progressive respiratory failure. There is a profound ascending paralysis commonly seen with MG, which was not observed in our patient. Another hallmark manifestation of MG is muscle weakness that is exacerbated by exertion and improves with rest [10]. This was clinically not observed nor reported by our patient during her hospital stay, effectively ruling out MG. GBS was also considered but much like MG, the pattern of paralysis is ascending which was not consistent with the patient's presentation [11]. For this distinct difference, we did not continue to consider GBS as a differential diagnosis. Finally, when considering Eaton Lambert syndrome, it is important to note symptoms such as muscle weakness, absent deep tendon reflexes, and autonomic dysfunction. Muscle weakness typically involves the proximal lower extremities, as patients frequently complain of experiencing difficulty when arising from the seated position [12]. Eaton Lambert syndrome is most commonly observed in patients with a confirmed diagnosis of small cell lung cancer. Our patient did not complain of the hallmark symptoms of Eaton Lambert syndrome and did not have any history of small cell lung cancer. A CT scan was not done for the diagnosis of lung cancer as it was low on our differential and carried a major risk to the fetus.

The significance of this case report is not only in the rarity of this disease, but its manifestation in a 25-year-old gravida 3, para 1 female, who was at 14 weeks and three days gestation. A preliminary diagnosis of wound botulism secondary to black tar heroin was rapidly considered given the symptoms of aspiration, facial droop, and respiratory failure requiring orotracheal intubation in a patient who was admitted to intramuscular injection of heroin. This method of intramuscular injection is favored amongst patients over IV injection because it avoids the telltale “track marks” [7]. In a case report of two pregnant women diagnosed with botulism, Ataee et al. mention how both patients were treated with antitoxin, with varying prognoses. One patient had muscle weakness that improved after five days and was discharged home; however, the second case required intubation and mechanical ventilation for 14 days total, with resultant discharge home. Ataee et al. emphasize that early intervention with botulism antitoxin correlates with improved prognosis in affected patients [1].

In a systematic review of 17 pregnant/postpartum women by Badell et al., the authors observed that more than half of the patients required intubation and mechanical ventilation, with two women deceased, and six infants born prematurely. This review did not yield any adverse effects to the mother or fetus associated with botulism antitoxin. Given the size of the antitoxin, which weighs approximately 150 kDa, it is too large to cross the placenta. Therefore, we believe the antitoxin should not have any short-term or long-term effects on the fetus and was therefore safely administered [9]. Although there is no clinical evidence of observed fetal harm, the safety profile of administering the antitoxin helped support our clinical decision when weighing the risks versus benefits to the mother and fetus. Ultimately in our case, in the acute setting, the decision for antitoxin administration was made.

Limitations to our case existed primarily due to its urgency and the patient’s non-compliance. Given the increased morbidity and mortality rates of this disease, a clinical diagnosis of botulism was made. Additional tests such as a Tensilon test for the confirmation of MG, a lumbar puncture to diagnose GBS, and electromyography to diagnose Eaton-Lambert were not completed due to low clinical suspicion [10-12]. ​Additionally, our patient was only at an estimated gestational age of 14 weeks and four days at the time that she left against medical advice, and she has not been to the hospital or our clinic for prenatal care since. In order to prevent the delay of treatment and poor outcomes, the decision was made for early administration of the antitoxin, raising concern of possible fetal harm. However, based on the limited published case reports we do not anticipate that the diagnosis of botulism and treatment with antitoxin will cause fetal harm. For these reasons, it is important for clinicians to maintain a high degree of suspicion for wound botulism in patients presenting with oculo-bulbar weakness who also use heroin.

## Conclusions

Botulism is a rare neuroparalytic illness caused by *C. botulinum* that can manifest as a descending flaccid paralysis. Wound botulism is majorly linked to the injection of BTH in the western United States. Although the symptomatology in pregnant and non-pregnant patients is similar, pregnant patients may experience a more severe course, and there is often diagnostic ambiguity due to a lack of pathognomonic features. Therefore, physicians should maintain a high clinical suspicion for botulism in pregnant patients presenting with neurological symptoms in the setting of BTH use.
